# LncRNA LASTR promote lung cancer progression through the miR-137/TGFA/PI3K/AKT axis through integration analysis

**DOI:** 10.7150/jca.66067

**Published:** 2022-01-06

**Authors:** Manhui Xia, Weibo Zhu, Chunmu Tao, Yuntian Lu, Feng Gao

**Affiliations:** 1 Department of Thoracic Surgery, Jingjiang People's Hospital of Jiangsu Province, Jingjiang City 214500, Jiangsu Province, China; 2 Department of Gastroenterology, Jingjiang People's Hospital of Jiangsu Province, Jingjiang City 214500, Jiangsu Province, China; 3 Department of Bioinformatics, Nantong University Medical School, Nantong, Jiangsu 226001, People's Republic of China

**Keywords:** LASTR, lncRNA, lung cancer, PI3K, miRNA

## Abstract

**Background:** Long noncoding RNAs (LncRNAs) are widely involved in the physiological and pathophysiological processes of cells. This study sought to identify novel lncRNAs that play key roles in progression of lung cancer.

**Methods:** Cells were purchased from the Cell Bank of Type Culture Collection of the Chinese Academy of Sciences. Public lung cancer data were retrieved from The Cancer Genome Atlas database. Statistical analyses were performed using SPSS, R and GraphPad Prism 8 software.

**Results:** Bioinformatic analysis showed that the lncRNA, LASTR (ENSG00000242147) was significantly upregulated in lung cancer tissues (LUAD and LUSC) compared with the expression level in adjacent normal tissue. Kaplan-Meier survival analysis showed that patients with higher LASTR expression level had a shorter overall survival and worse clinical features relative to patients with low LASTR expression levels. qRT-PCR results showed that LASTR was highly expressed in lung cancer cell lines relative to the expression level in normal lung epithelial cell line. Cell phenotype experiments indicated that knockdown of LASTR significantly inhibited proliferation and metastatic ability of lung cancer cells. Analysis of the downstream mechanism of LASTR demonstrated that LASTR exerts the oncogene effect through the miR-137/TGFA axis. GSEA results indicated that LASTR exhibits its activity by activating the PI3K/AKT signaling pathway, which was validated by western blotting assay.

**Conclusion:** In summary, the results of the present study showed that LASTR promotes lung cancer progression through miR-137/TGFA/PI3K/AKT axis.

## Introduction

Lung cancer is the leading cause of cancer mortality worldwide. Lung cancer contributes to the highest cancer incidences globally with 2.2 million new cases and 1.8 million deaths reported in 2020 1. Lung cancer is classified into two major histological subtypes based on the histopathological presentation: small cell lung cancer (SCLC) and non-SCLC (NSCLC). NSCLC accounts for about 85% of all lung cancer cases 2. NSCLC is a highly heterogeneous disease, characterized by high mutational burden and complex tumor microenvironment thus treatment with generalized clinical approach is not effective 3. The 5-year overall survival for lung cancer is less than 5% despite significant advances in development of diagnostic modalities and standard treatments for lung cancer patients 4. Therefore, it is essential to explore more effective targets and develop novel strategies for improving prognosis prediction and for development of more effective lung cancer therapies.

Long Noncoding RNAs (lncRNAs) are located in the nucleus or cytoplasm and are generally defined as noncoding RNA (ncRNA) sequences comprising more than 200 nucleotides 5. Previous studies report that lncRNAs play key roles in multiple cellular and biological processes, including cell growth, development, tumor initiation, progression and metastasis 6. Moreover, studies report that several lncRNAs are attractive targets for therapeutic intervention of various cancer types 7, 8. For example, LINC02418 is significantly overexpressed in colorectal cancer tissues and plays an important role in CRC tumorigenesis through the LINC02418/miR-1273g-3p/MELK axis 9. In addition, expression of the lncRNA, MACC1-AS1 is upregulated under metabolic stress, thus inducing a malignant phenotype in gastric cancer cells 10. Furthermore, a novel oncogenic lncRNA, LINC00673 significantly promotes tumorigenesis ability of A549 cells *in vivo* by sponging miR-150-5p 11. These findings indicate that lncRNAs are promising targets in different cancers thus it is imperative to explore the clinical value of lncRNAs in lung cancer.

In the present study, the novel lncRNA, LASTR was identified through transcriptome analysis. Bioinformatic analysis showed that LASTR is highly expressed in lung cancer tissue and is associated with worse clinical outcomes. *In vitro* experiments showed that LASTR significantly promoted proliferation and cell metastatic ability of lung cancer cells. Further analysis showed that LASTR exerted an oncogene effect through miR-137/TGFA axis and the downstream target pathway was the classical PI3K/AKT pathway. These results indicated that LASTR promotes lung cancer progression and might be a novel tumor marker of lung cancer.

## Methods

### Public data analysis

RNA-seq data for lung cancer patients were retrieved from TCGA-GDC webserver. Data comprised 108 normal samples and 1137 LUAD and LUSC tumor samples. For the open-accessed data of TCGA, the inclusion criteria of patients enrolled in this study was: 1. the patients with complete clinical information (survival information, stage, age, grade); 2. the patients with transcriptomic data. The exclusion criteria was: 1. the patients with incomplete clinical information and transcriptomic data. Corresponding clinical information was directly retrieved from TCGA-GDC in “bcr-xml” file format. All the data were subjected to a series of pre-processing steps including normalization, probe annotation and missing value completion, before analysis. Survival analysis and clinical correlation analysis were conducted using survival package in R software. Gene set variation analysis (GSVA) was conducted to explore enriched biological pathways between high and low LASTR samples in the TCGA database using the Hallmark gene set. The parameters of patients enrolled into the current study were shown in ***Table 1***.

### Cells, tissue and quantitative real-time PCR (qRT-PCR)

A normal human lung epithelial cell line (BEAS-2B) and four lung cancer cell lines (A549, H1650, H1299 and H1975) were purchased from the Cell Bank of Type Culture Collection of the Chinese Academy of Sciences. Cells cultured under 37°C and 5% CO_2_ with complete medium. Lung cancer tissues (pathologically confirmed) used in this study were obtained from Jingjiang People's Hospital of Jiangsu Province and all the patients signed an informed consent form prior to the study. The present study was approved by the ethics committee of Jingjiang People's Hospital of Jiangsu Province.

Total RNA was extracted using TriZol reagent according to manufacturer's instructions. RNA was then reverse transcribed to obtain cDNA. The SyBr Green PCR system was used to perform qRT-PCR (Toyobo). Primers used were as follows: LASTR; forward primer: 5'-AGTGGGTGAAGTCCTGGTT-3', reverse primer: 5'-GGCTGAAGGGTTTAGATG-3'; TGFA; forward primer: 5'-AGGTCCGAAAACACTGTGAGT-3', reverse primer: 5'-AGCAAGCGGTTCTTCCCTTC-3'. miR-137; RT primer, 5'-GTCGTATCCAGTGCAGGGTCCGAGGTATTCGCACTGGATACGACCTACGC-3', forward primer: 5'-GGTCGTGGTTATTGCTTAAGAATAC-3', reverse primer: 5'-CAGTGCAGGGTCCGAGGT-3'.

### Cell transfections

Plasmids for LASTR short hairpin RNA (sh-LASTR), sh-TGFA and sh-NC were purchased from Obio Technology (Shanghai). The NC group were control group and were cells that transfected with scrambled siRNA. miR-137 inhibitor and mimics were purchased from Gene Pharma (Suzhou, China). Cells were seeded into a six-well plate at equal density before transfections. Lipofectamine 2000 transfection kits were used to perform cell transfections according to the manufacturer's protocol. Lipofectamine 2000 and plasmids were added into the wells at a ratio of 1:1. The supernatant was discarded after 12h and fresh medium was added into the wells and incubated. The sequence used for LASTR knockdown were as follows: shRNA1: 5'-GGAAATTCAGATCATCTAAAC-3', shRNA2: 5'-AGGGTTAATGACTCAATTTTT-3', shRNA3: 5'-TGCTAGTAATGACAATCATGT-3'.

### Western blotting analysis

Cells were cultured to 100% confluency and RIPA reagent (1% phosphatase inhibitor and 1% protease inhibitor) was added. Cells were lysed on ice for 1.5h and frozen at -80 °C overnight. Extracted total protein samples were mixed with loading buffer and boiled for 20 min. SDS-PAGE was then performed for separation of proteins. Proteins were then transferred to a PVDF membrane and incubated with primary antibodies. Primary antibodies (anti-AKT, anti-p-AKT, anti-cyclinD1, anti-β-Actin) were purchased from the Cell Signaling Technology (CST).

### Colony formation assay

Transfected cells were inoculated into a six-well plate at a concentration of 500 cells per well. Cells were then incubated in a medium supplemented with 10% FBS which was replaced every four days. Cells were incubated for 12 days, fixed using anhydrous ethanol and stained with 0.1% crystal violet.

### 5-Ethynyl-2'-deoxyuridine (EdU) assay

EdU assay was performed using EdU staining kit (RiboBio). Cells were seeded into a six-well plate at a density of 2×10^5^ cells per well. EDU reaction solution was added to the medium after cells adhered to the wall according to the manufacturer's protocol. DAPI solution was used for nucleus staining. Fluorescence analysis was performed using an inverted fluorescence microscope.

### CCK8 assay

Cells were seeded into a 24-well plate at a density of 1000 cells per well. CCK8 reagent was added into the wells and cells cultured for 1.5 hours. The absorbance was determined at 450nm.

### Transwell assay

The lower chamber was comprised 600ul of medium supplemented with 20% FBS. 200ul serum-free conditioned medium with 2*10^4^ cells was added to the upper chamber. Cells were incubated for 24h then fixed with 4% paraformaldehyde and stained with crystal violet.

### Wound healing assay

Transfected cells were seeded into a six-well plate and incubated to achieve 100% confluency. A 200ul tip was used to make a cell scratch on the plates. Cell motility was observed at 0h and 24h time points.

### Luciferase reporter gene assay

Luciferase reporter vectors were co-transfected with miR-137 mimics using lipofectamine 3000. Transfected cells were incubated for 48h then lysed. psiCHECK-2-LASTR-wild-type (LASTR -wt), psiCHECK-2- LASTR -mutant (LASTR -mut), psiCHECK-2-TGFA-wild-type (TGFA -wt) and psiCHECK-2- TGFA -mutant (TGFA -mut) vectors were created using PCR methods and coloned into plasmids (Invitrogen, Thermo Fisher Scientific, Inc.). Dual luciferase assay was performed using dual-Luciferase Assay kit (Promega) using the cell lysates. Relative luciferase activity was calculated as the ratio of firefly luciferase activity to Renilla luciferase activity.

### Biotin RNA pulldown assay

RNA pulldown assay was conducted according to the protocol of PierceTM Magnetic RNA-protein Pull-Down kit (20164Y) purchased from Thermo Fisher Scientific. Biotin-labeled LASTR probe and anti-probe were incubated with cell lysate for 1 h. Streptavidin-coupled agarose beads (Invitrogen) was used to pull down the binding complex. The probes included: biotin-LASTR, 5'-AGTGAAGGGCTGAAGGGTTTAG-3'; anti-LASTR, 5'- AAGAGAGAAGACAGTGGGTGAAGT-3'; biotin-miR-137, 5'-ATTATCCACCCAAGAATACCCGT-3'; anti-miR-137, 5'-ACGGGTATTCTTGGGTGGATAAT-3'.

### Fluorescence in situ hybridization (FISH) assay

The fluorescence-labeled probes of LASTR (5'-TATTGCTTGGATGTGAGTCTCTGA-3') was designed and synthesized in Jima Com(Shanghai, China). FISH assay was performed according to the protocol of Ribo FISH Kit (RiboBio, Guangzhou, China). Cell climbing slices were observed and photographed with con-focal laser scanning microscope (Leica TCS SP2).

### Statistical analysis

All experiments were performed in three biological replicates each with three technical replicates. SPSS, R and GraphPad Prism 8 software were used for statistical analysis. Student t-test was used to compare differences between the cancer group and the normal group. P value < 0.05 (two-sided) was considered statistically significant.

## Results

### LASTR is upregulated in lung cancer tissues and is associated with poor clinical features

**Figure 1:**
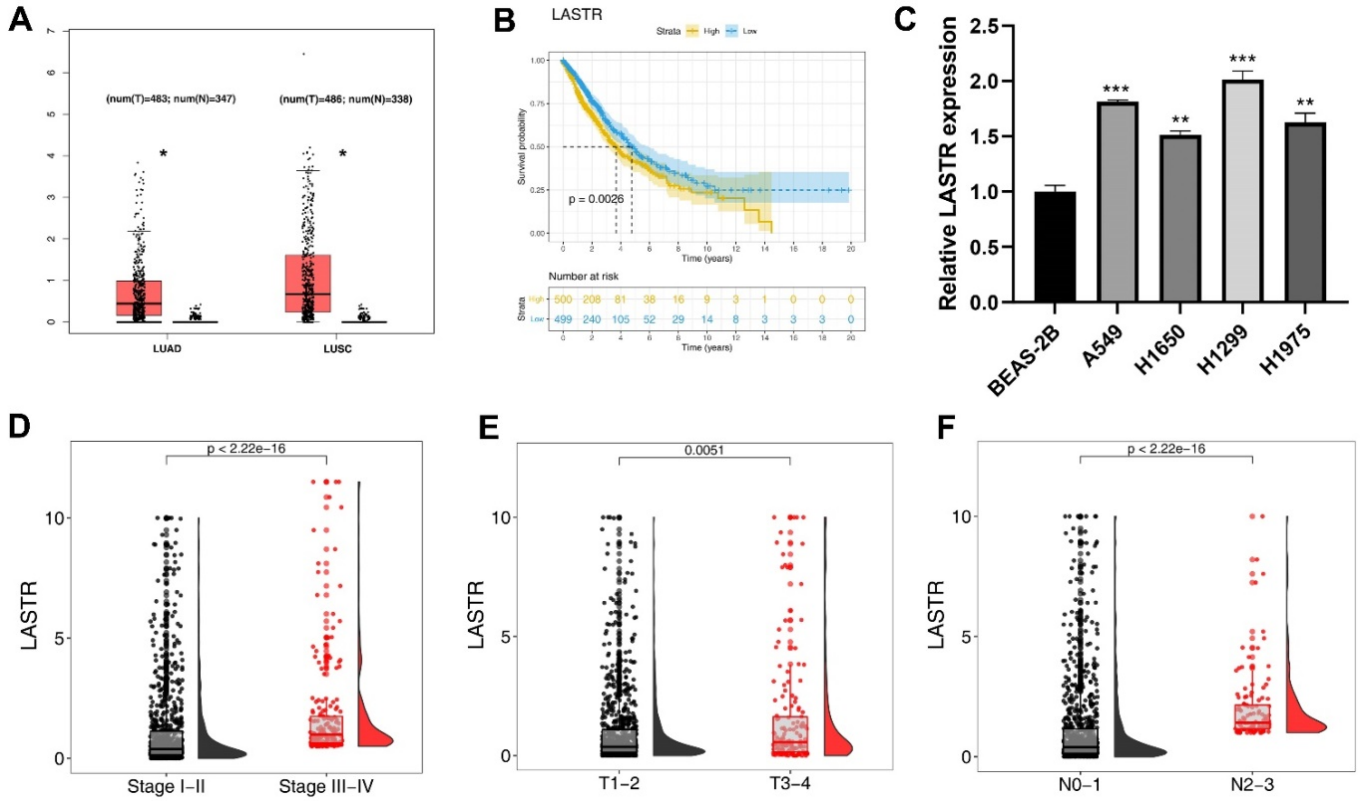
** LncRNA LASTR was upregulated in lung cancer and associated with poor clinical features. Notes: A:** TCGA data showed that the lncRNA LASTR had a higher expression level in the LUAD and LUSC tumor tissue, * = P < 0.05; **B:** KM survival curve showed that the patients with high LASTR expression tend to have a shorter survival time; **C:** LASTR was upregulated in four lung cancer cell lines compared with normal lung epithelial cell line, ** = P < 0.01, *** = P < 0.001; **D-F:** LASTR was correlated with worse clinical features, including clinical stage, T and N classification. **Abbreviations:** TCGA, The Cancer Genome Atlas; LUAD, lung adenocarcinoma; LUSC, lung squamous cell carcinoma; KM, Kaplan-Meier

The expression pattern of LASTR was explored in TCGA-LUAD and LUSC tissues. The results showed a higher expression level of LASTR in tumor tissue compared with the expression level in corresponding normal tissues (***Figure 1A***). Kaplan-Meier survival curves showed that patients with high LASTR expression level had a shorter survival time relative to patients with low LASTR expression level (***Figure 1B***, P < 0.05). Gene expression analysis using cell lines showed that LASRT was highly expressed in four cancer cell lines relative to the expression level in the normal lung epithelial cell line (***Figure 1C***). Clinical correlation analysis demonstrated that LASTR expression level was correlated with worse clinical features, including clinical stage, T classification and N classification (***Figure 1D-F***).

### LASTR promotes proliferation and invasion of lung cancer cells

**Figure 2:**
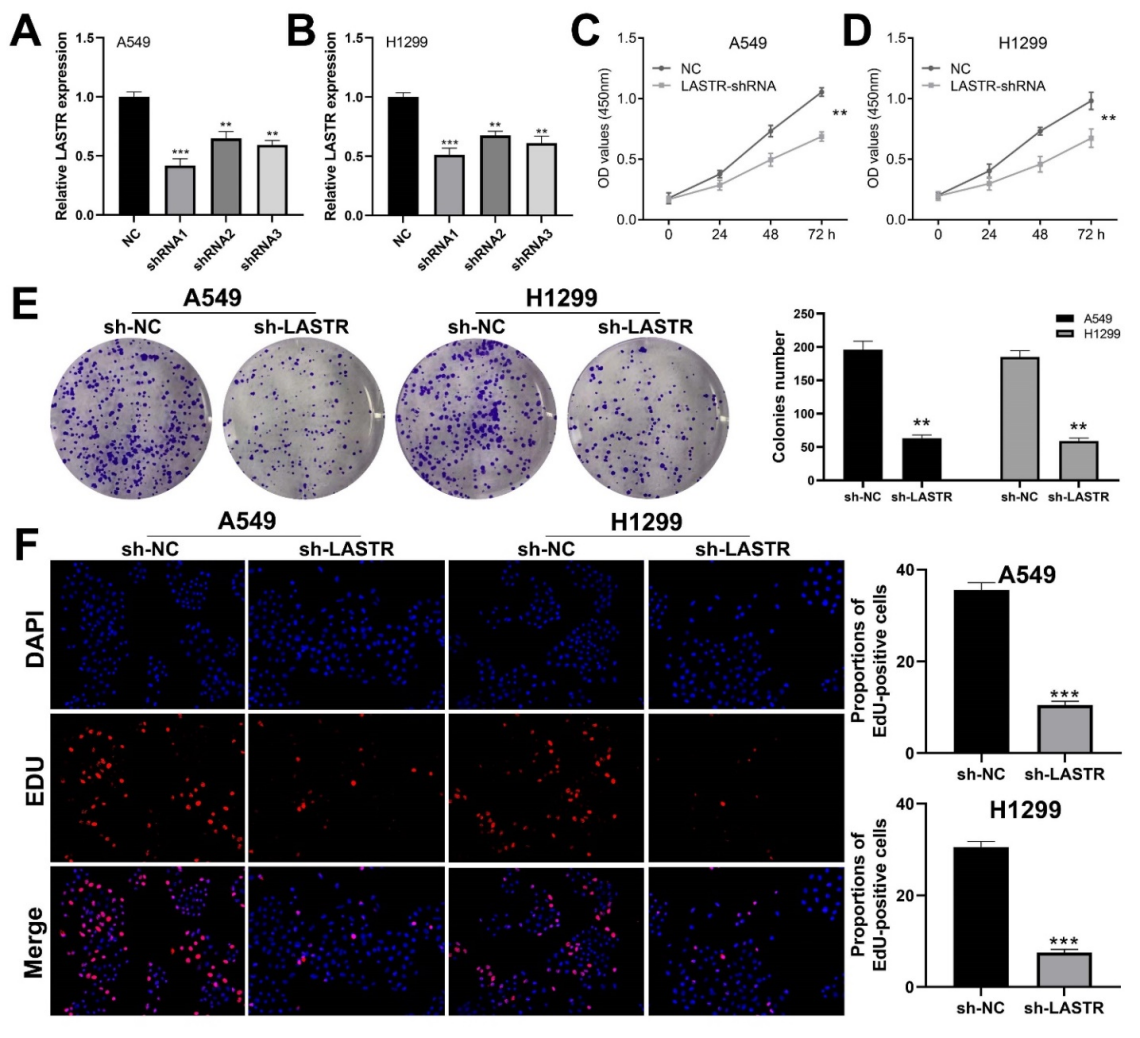
** LASTR promotes lung cancer cells proliferation. Notes: A-B:** qRT-PCR was used to assess the knockdown efficiency of LASTR in the A549 and H1299 cell line and the shRNA1 had the best efficiency, which was selected for further experiments, ** = P < 0.01, *** = P < 0.001; **C-D:** CCK8 assay showed that the silencing of LASTR significantly inhibits cell proliferation of lung cancer cells, ** = P < 0.01;** E:** Colony formation assay demonstrated that the knockdown of LASTR remarkably reduces the colony number, ** = P < 0.01; **F:** Edu assay indicated that knockdown of LASTR remarkably increased the proportion of EdU-positive cells, *** = P < 0.001.

**Figure 3:**
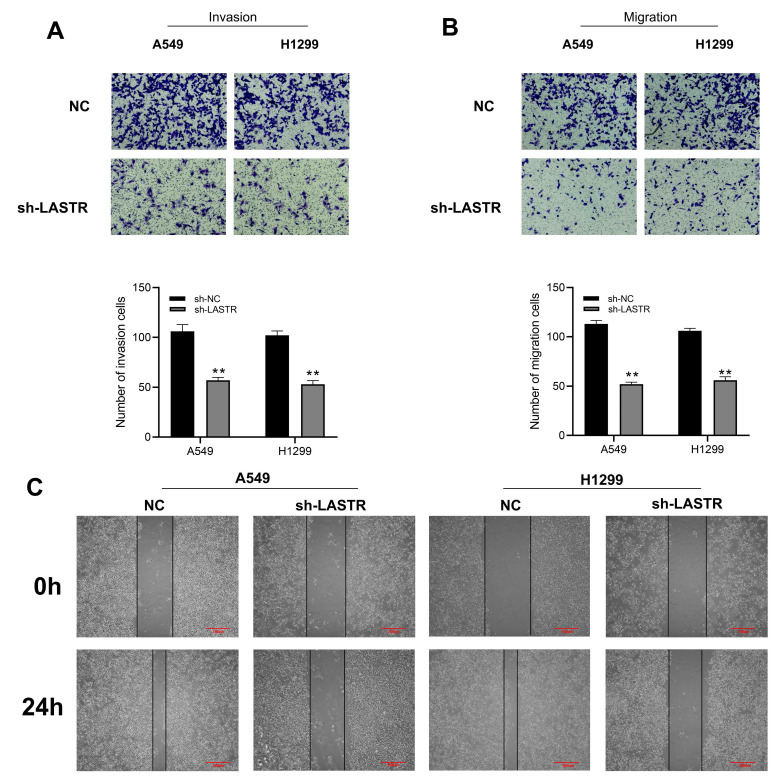
** LASTR facilitate the metastatic potential of lung cancer cells. Notes: A-B:** Transwell assay showed that the cells with LASTR knockdown had lower migration and invasion ability, ** = P < 0.01; **C:** Wound-healing assay demonstrated that the silencing of LASTR significantly inhibits lung cancer cell movement.

Cell phenotype experiments were performed using control and LASTR knockdown groups to further explore the biological role of LASTR in lung cancer. Efficacy of LSATR knockdown was validated by qRT-PCR analysis (***Figure 2A-B***). CCK8 assay showed that the control wells had a higher absorbance, indicating that downregulation of LASTR expression decreased the proliferation ability of lung cancer cells (***Figure 2C-D***). In addition, colony formation assay showed a decrease in colony formation after downregulation of LASTR expression (***Figure 2E***). Furthermore, EdU assay indicated that knockdown of LASTR remarkably increased the proportion of EdU-positive cells (***Figure 2F***). The underlying effect of LASTR on cell metastatic potential was then explored. Transwell assay results indicated that knockdown of LASTR significantly decreased invasion and migration ability of cancer cells relative to control cells (***Figure 3A-B***). Moreover, wound-healing assay showed that the cells with low expression levels of LASTR moved slower compared with the control cells (***Figure 3C***).

### LASTR targets miR-137 and to modulate progression of lung cancer

**Figure 4:**
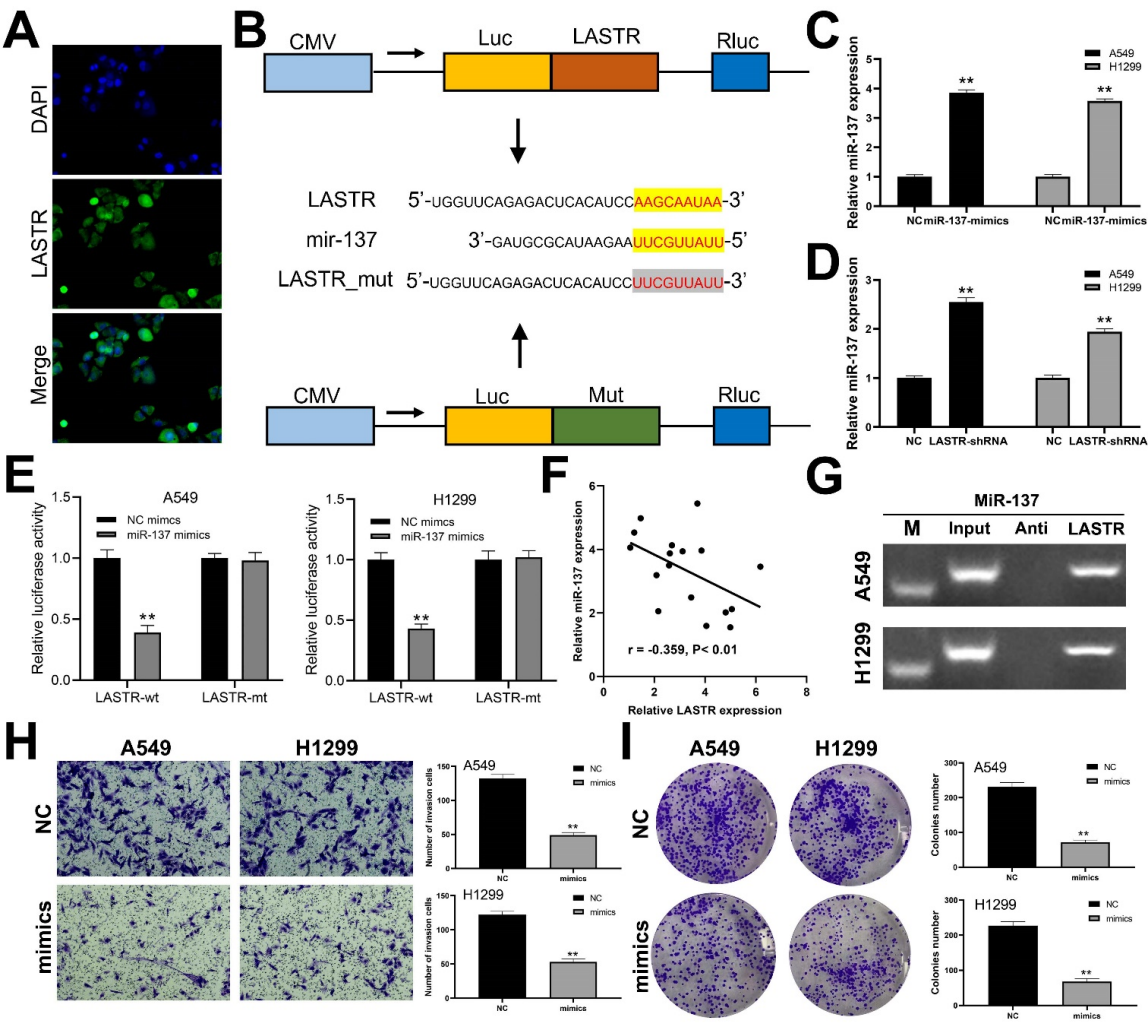
** miR-137 is a target miRNA of LASTR. Notes: A:** FISH assay was performed to explore the subcellular localization of LASTR. **B:** The binding sequence of miR-137 and LASTR predicted by LncBase and the sequence mutated for the luciferase reporter assay; **C:** qRT-PCR showed a satisfactory overexpressed efficiency of miR-137 in A549 and H1299 cell lines, ** = P < 0.01; **D:** qRT-PCR was conducted to assess the miR-137 change in the control and sh-LASTR cells, ** = P < 0.01; **E:** Luciferase reporter assay was performed in the cells with wild-type 3'-UTR and mut-3'-UTR of LASTR, ** = P < 0.01; **F:** A negative linear correlation between miR-137 and LASTR was also found based on 17 lung cancer tissue; **G:** RNA pulldown assay showed that LASTR could bind to more miR-137 relative to its anti-probe; **H:** Transwell assay was performed in the control and sh-LASTR cells, ** = P < 0.01; **I:** Colony formation assay was performed in the control and sh-LASTR cells, ** = P < 0.01. **Abbreviations:** FISH: fluorescence in situ hybridization

We further explored the subcellular localization of LASTR in cells and found that LASTR was mainly localized in the cytoplasm, indicating it may act through a competitive endogenous RNA (ceRNA) mechanism (***Figure 4A***). Analysis using LncBase v2.0 database (http://carolina.imis.athena-innovation.gr/diana_tools/web/index.php?r=lncbasev2%2Findex) showed that miR-137 is the target miRNA for LASTR. The binding mechanism between LASTR and miR-137 is shown in ***Figure 4B***. Further analysis was performed to explore whether LASTR directly binds to miR-137. Mimics of miR-137 were transfected into A549 and H1299 cells and the transfection efficiency was validated by qRT-PCR (***Figure 4C***). Significant overexpression of miR-137 was observed in LASTR knockdown cells, relative to the expression level of the control cells (***Figure 4D***). Luciferase reporter assays indicated that overexpression of miR-137 significantly inhibited the luciferase activities in A549 and H1299 cells with wild-type 3'-UTR of LASTR, whereas overexpression of miR-137 had no effect on mut-type 3'-UTR of LASTR in A549 and H1299 cells (***Figure 4E***). A negative correlation was observed between miR-137 and LASTR was observed at the tissue level based on analysis of 17 lung cancer tissues (***Figure 4F***; r = -0.359, P < 0.01). RNA pulldown assay showed that the LASTR probe could enrich more miR-137 compared with its anti-probe (***Figure 4G***). Furthermore, the role of miR-137 in lung cancer was explored. Transwell assay demonstrated that overexpression of miR-137 significantly decreased invasion ability of A549 and H1299 cells (***Figure 4H***). In addition, downregulation of miR-137 significantly inhibited proliferation of lung cancer cells (***Figure 4I***).

### TGFA targets miR-137 to exert its activity in lung cancer

**Figure 5:**
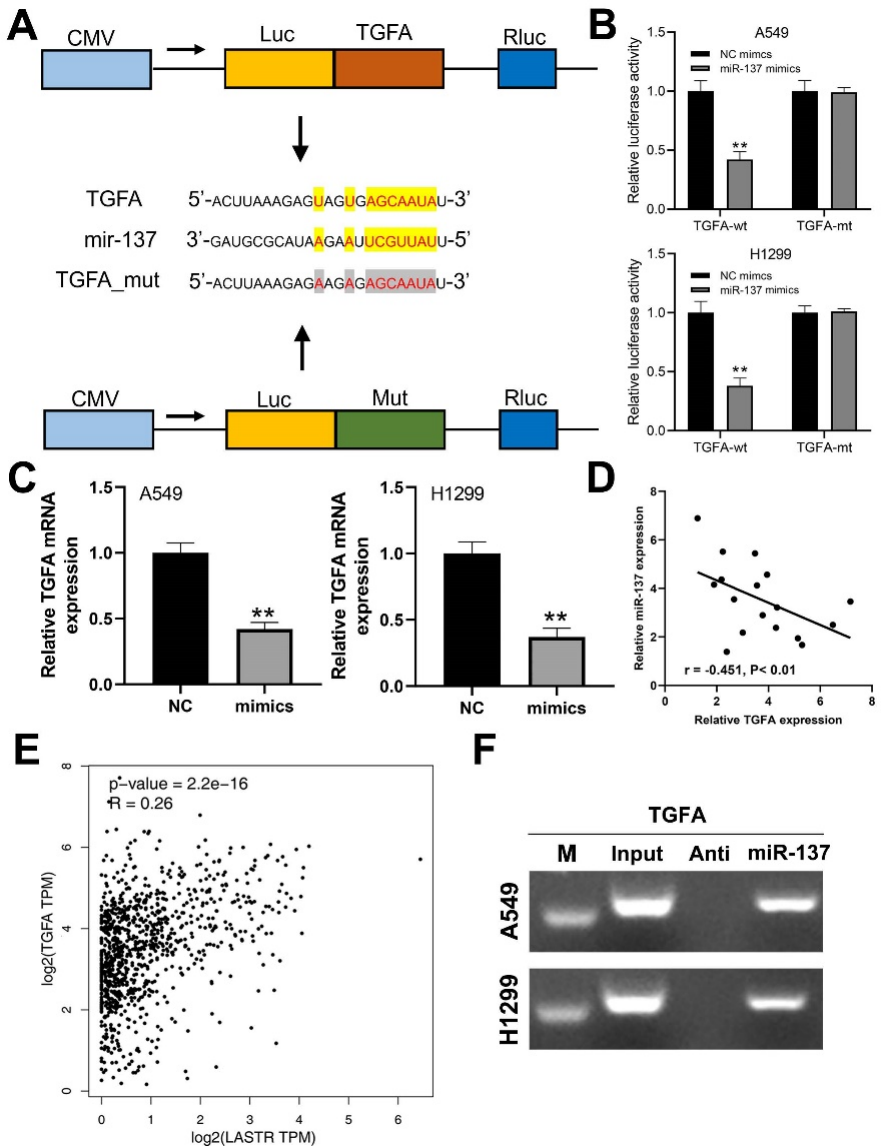
** TGFA is a target gene of miR-137. Notes: A:** The binding sequence of TGFA and miR-137 predicted by LncBase and the sequence mutated for the luciferase reporter assay; **B:** Luciferase reporter assay was performed in the cells with wild-type 3'-UTR and mut-type 3'-UTR of TGFA, ** = P < 0.01; **C:** qRT-PCR was conducted to assess the TGFA change in the control and miR-137-mimics cells, ** = P < 0.01; **D:** A negative linear correlation between miR-137 and TGFA was also found based on 17 lung cancer tissue; **E:** A positive correlation between LASTR and TGFA was found based on the TCGA database (GEPIA website);** F:** RNA pulldown assay showed that miR-137 could bind to more TGFA relative to its anti-probe.

A previous study has reported that TGFA gene promotes progression of lung cancer cells. In the present study, prediction using Starbase database (http://starbase.sysu.edu.cn/panCancer.php) showed that TGFA is a potential target gene in lung cancer (***Figure 5A***). Luciferase reporter assay was performed to explore the binding between miR-137 and TGFA. A significant decrease in luciferase activities in wild-type 3'-UTR of TGFA cells was observed when miR-137 was overexpressed, but this decrease in luciferase activities was not observed in the mut-type 3'-UTR cells (***Figure 5B***). Moreover, mRNA expression level of TGFA was significantly decreased in cells overexpressing miR-137 (***Figure 5C***). In addition, qRT-PCR results of based on tissues indicated a significant negative correlation between miR-137 and TGFA mRNA level (***Figure 5D***; r = -0.451, P < 0.01). A positive correlation was observed between LASTR and TGFA expression level based data for lung cancer patients retrieved from the TCGA database (***Figure 5E***, r = 0.26, P < 0.001). RNA pulldown revealed that the miR-137 could bind to more TGFA compared with its anti-probe, indicating the specific interaction between miR-137 and TGFA (***Figure 5F***).

### LASTR exerts an oncogene effect through miR-137/TGFA/PI3K/AKT axis in lung cancer

**Figure 6:**
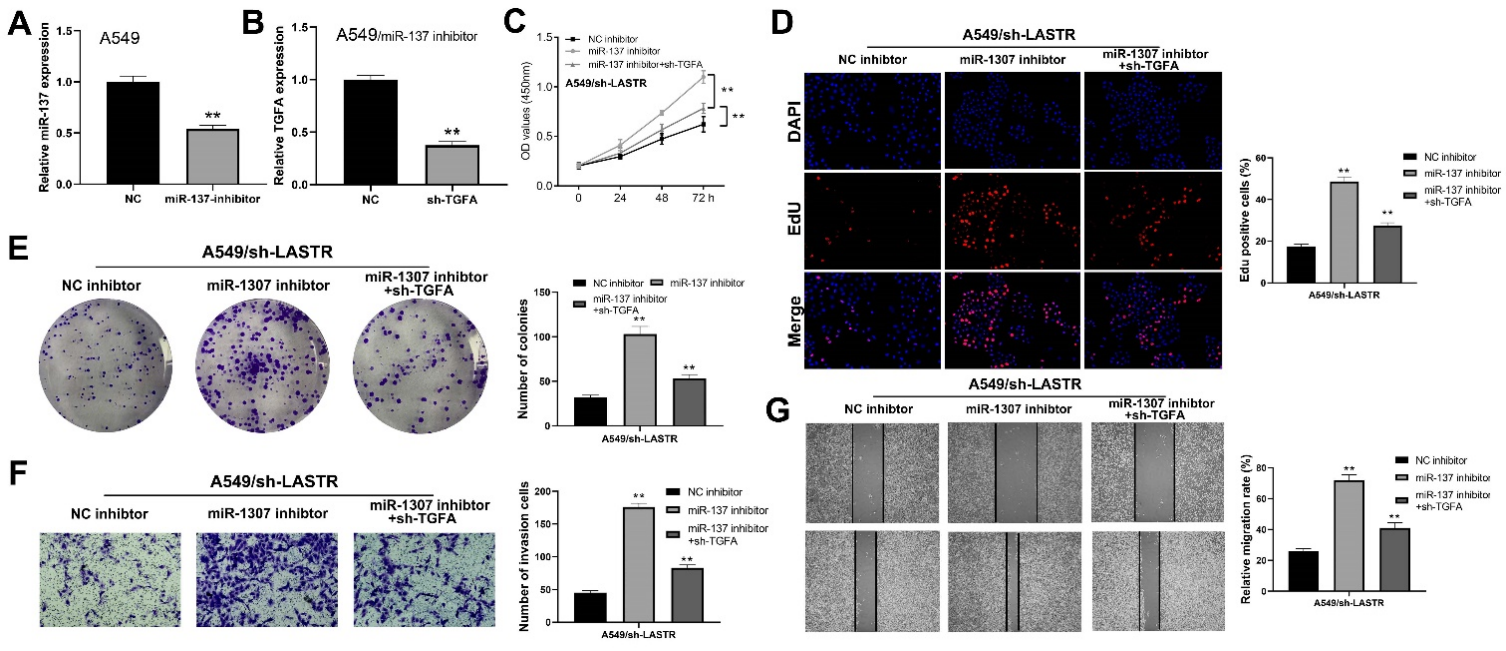
** LncRNA LASTR enhances lung cancer progression by regulating the miR-137/TGFA axis. Notes: A:** qRT-PCR showed a satisfactory miR-137 inhibitor efficiency in A549 cell line; **B:** qRT-PCR showed a satisfactory TGFA knockdown efficiency in A549/miR-137 inhibitor cell line** C-E:** CCK8 (C), EdU (D) and colony formation (E) assays were performed in the cells treated by sh-LASTR, sh-LASRT+miR-137 inhibitor and sh-LASRT+miR-137 inhibitor+sh-TGFA to explore the effect on cell proliferation, ** = P < 0.01; **F-G:** Transwell (F) and wound-healing assays (G) were performed in the cells treated by sh-LASTR, sh-LASRT+miR-137 inhibitor and sh-LASRT+miR-137 inhibitor+sh-TGFA to explore the impact on cell metastatic potential, ** = P < 0.01.

**Figure 7:**
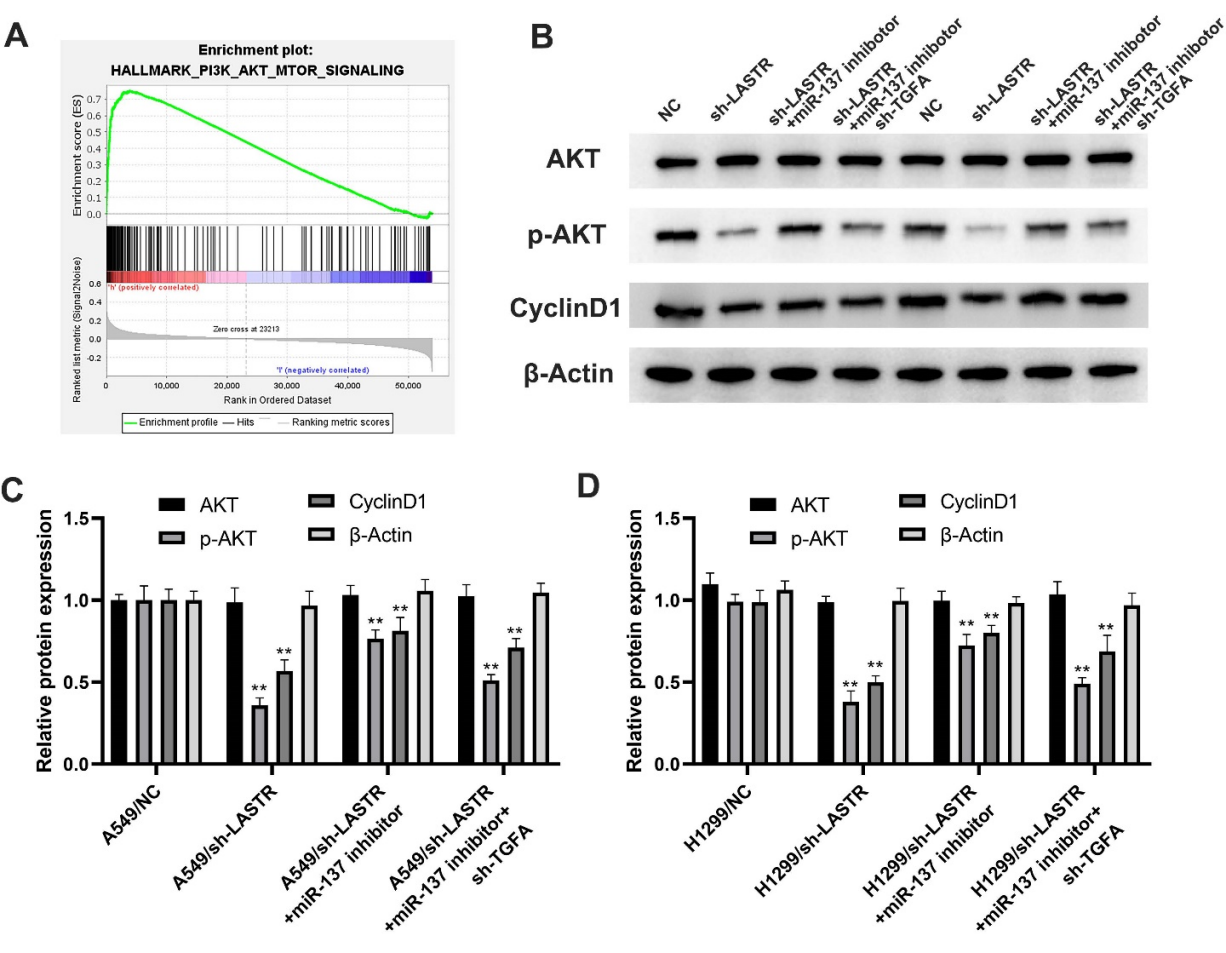
** PI3K/AKT signaling pathway is the downstream pathway of LASTR/miR-137/TGFA axis. Notes: A:** GSEA analysis was performed to explore the underlying cancer pathway involved in LASTR and showed that PI3K/AKT patwhay was the top enriched pathway; **B-D:** The changes of key marker in PI3K/AKT pathway was validated by western blotting assay in different treated cells (control, sh-LASTR, sh-LASRT+miR-137 inhibitor and sh-LASRT+miR-137 inhibitor+sh-TGFA). **Abbreviations:** GSEA, gene set enrichment analysis

Further analysis was conducted to explore whether the lncRNA LASTR promotes lung cancer progression through a ceRNA mechanism (LASTR/miR-137/TGFA axis). miR-137 was knocked down in cells with downregulated LASTR expression (***Figure 6A***). Then, TGFA was knocked down in miR-137 inhibitor cells (***Figure 6B***). CCK8 assay showed that inhibition of miR-137 promoted proliferation of LASTR knockdown cells, which was antagonized by downregulation of TGFA expression (***Figure 6C***). EdU assay indicated that knockdown of miR-137 significantly increased the proportion of EdU-positive cells, whereas downregulation of TGFA alleviated this effect (***Figure 6D***). In addition, colony formation assay demonstrated that cells transfected with miR-137 inhibitor and sh-TGFA plasmid formed more colonies compared with the cells treated with miR-137 inhibitor (***Figure 6E)***. Moreover, transwell and wound-healing assays showed consistent results with the cell proliferation assay (***Figure 6F-G***). These results indicated that LASTR promotes lung cancer progression by modulating the downstream miR-137/TGFA axis. GSVA analysis was performed to explore the potential biological pathways associated with LASTR activity. The findings showed that the most significantly enriched pathway was PI3K/AKT pathway, thus it was selected for further validation (***Figure 7A***). Western blotting assay showed that knockdown of LASTR significantly inhibited activation of PI3K/AKT pathway. Notably, treatment of cells with miR-137 inhibitor partially alleviated this effect, whereas downregulation of TGFA inhibited PI3K/AKT pathway in miR-137 knockdown cells (***Figure 7B-D***). These results indicated that LASTR exhibits oncogene effects in lung cancer by modulating the miR-137/TGFA/PI3K/AKT axis.

## Discussion

Lung cancer is a highly aggressive malignancy with high incidence and mortality worldwide. However, the five-year survival rates of lung cancer have improved from 17.2% reported 10 years ago to 21.7% 12. Epidemiologic studies report significant association between smoking and lung carcinogenesis 13. The prognosis of advanced lung cancer patients is still unsatisfactory despite advances in lung cancer treatment 2. Therefore, it is imperative to discover promising prognostic markers and therapeutic targets to improve the clinical outcomes of lung cancer patients.

Recent studies reported that lncRNAs play important roles in cancer development through regulatory effects on RNA and protein levels 14. For instance, the lncRNA GAS5 inhibits progression of gastric cancer by interacting with and triggering YAP phosphorylation 15. PCA3, a lncRNA regulated by a steroid receptor is overexpressed in most prostate cancer patients and is detected in the urine of these cancer patients 16. Moreover, lncRNAs are directly involved in cell transcription by interacting with specific transcribing complexes or cis-acting elements, thus affecting cell processes 17, 18. Moreover, lncRNAs can regulate cell signaling to affect the tumor cell biological behavior. Liu et al. reported that the lncRNA, NKILA interacted with NF-κB/IκB complex and inhibited phosphorylation of IκB medicated by IKK 19. Schmitt et al. observed that the lncRNA, DINO stabilizes the p53 protein by physically interacting with it thus promoting cell DNA damage 20. The findings of the present study showed that the lncRNA, LASRT promotes lung cancer progression and is a potential novel tumor marker for clinical diagnosis and treatment of lung cancer.

CeRNA mechanism and the PI3K/AKT pathway have been widely investigated in different cancers 21, 22. Luan et al. demonstrated that the lncRNA, XLOC_006390 promotes cervical cancer tumorigenesis and metastasis through a ceRNA mechanism by targeting miR-331-3p and miR-338-3p 23. Moreover, Chen et al. reported that the lncRNA, OIP5-AS1 exerts its cancer-promoting effect in cervical cancer by acting as miR-143-3p sponge and upregulating SMAD3 expression 24. Gao et al. explored the role of the PI3K/AKT pathway in lung cancer and revealed that lung cancer patients expressing both PD-L1 and IFN-γ have a better prognosis. Analysis showed that the underlying mechanism was induction of the activation of PI3K/AKT pathway by IFN-γ 25. Furthermore, PI3K/AKT pathway may be a downstream mechanism involved in brain metastasis of lung cancer 26. The findings of the current study showed that the lncRNA, LASTR promotes lung cancer cell progression through a ceRNA mechanism (miR-137/TGFA axis). LASTR thus modulates PI3K/AKT downstream pathway to affect the regulatory network of lung cancer.

This is the first study to explore the role of LASTR in lung cancer. In the present study, the novel lncRNA, LASTR was identified through bioinformatics analysis based on publicly available data. The results indicated that LASTR was highly expressed in lung cancer tissue and high expression level was associated with poor clinical features. qRT-PCR results showed that lung cancer cell lines had higher LASTR expression level relative to normal lung epithelial cell lines. Results from *in vitro* experiments revealed that LASTR significantly promotes proliferation and invasion of lung cancer cell lines. Further analysis showed that miR-137 is a target of LASTR, which can be targeted to inhibit proliferation of lung cancer cells. Moreover, analysis was performed to explore whether LASTR exerts its oncogene effect through a ceRNA mechanism. The findings indicated that miR-137/TGFA is a downstream axis for LASTR activity in lung cancer. Western blotting assay showed that the LASTR/miR-137/TGFA axis modulates activity of the classical PI3K/AKT signaling pathway to promote proliferation of lung cancer cells. The findings of the present study showed that the lncRNA, LASTR plays an oncogene role in lung cancer through miR-137/TGFA/PI3K/AKT axis.

## Conclusion

A novel lncRNA, LASTR was identified in lung cancer. LASTR was highly expressed in lung cancer tissues and cells and high expression level was associated with poor clinical features. Subsequent experiments showed that the LASRT significantly promotes lung cancer progression through the miR-137/TGFA/PI3K/AKT axis.

## Figures and Tables

**Table 1:** Clinical parameters of patients enrolled in the study

		TCGA-LUAD		TCGA-LUSC	
Clinical features		Number of Patients (n)	Accounting for (%)	Number of Patients (n)	Accounting for (%)
Age	<65	223	42.7	170	33.7
	>=65	280	53.6	325	64.5
	Unknow	19	3.6	9	1.8
Gender	Male	242	46.4	373	74.0
	Female	280	53.6	131	25.9
Stage	Stage I	279	53.4	245	48.6
	Stage II	124	23.8	163	32.3
	Stage III	85	16.3	85	16.9
	Stage IV	26	4.9	7	1.4
	Unknow	8	1.5	4	0.8
T-stage	T1	172	32.9	114	22.6
	T2	281	53.8	295	58.5
	T3	47	9.0	71	14.1
	T4	19	3.6	24	4.8
	Unknow	3	0.6	0	0
N-stage	N0	335	64.2	320	63.5
	N1	98	18.8	133	26.4
	N2	75	14.4	40	7.9
	N3	2	0.4	5	0.9
	Unknow	12	2.3	6	1.1
M-stage	M0	353	67.6	414	82.1
	M1	25	4.8	7	1.4
	Unknow	144	27.6	83	16.5

## Abbreviations

LUAD: lung adenocarcinoma
LUSC: lung squamous cell carcinoma
SCLC: small cell lung cancer
NSCLC: non-small cell lung cancer
lncRNA: long noncoding RNA
ncRNA: noncoding RNA
TCGA: The Cancer Genome Atlas
GSVA: Gene set variation analysis
